# Neutrophil to Lymphocyte Ratio and Platelet to Lymphocyte Ratio in Poststroke Depression: A Systematic Review and Meta-Analysis

**DOI:** 10.1155/2022/5911408

**Published:** 2022-08-08

**Authors:** Shirin Sarejloo, Erfan Abadifard, Zhian Jamal Othman, Fatemeh Zafarani, Monireh Khanzadeh, Saeed Sadigh-Eteghad, Fereshteh Farajdokht, Asghar Mohammadpoorasl, Shokoufeh Khanzadeh

**Affiliations:** ^1^Cardiovascular Research Center, Shiraz University of Medical Sciences, Shiraz, Iran; ^2^Students' Scientific Research Center (SSRC), Tehran University of Medical Sciences, Tehran, Iran; ^3^Department of Physical Education and Sport Sciences, Cihan University, Erbil, Iraq; ^4^Student Research Committee, Tabriz University of Medical Sciences, Tabriz, Iran; ^5^Neurosciences Research Center, Tabriz University of Medical Sciences, Tabriz, Iran; ^6^Department of Statistics and Epidemiology, Faculty of Health, Tabriz University of Medical Sciences, Tabriz, Iran

## Abstract

**Objectives:**

Evidence shows that stroke-induced inflammatory responses play an essential role in the development of poststroke depression (PSD). The goal of this systematic review and meta-analysis was to critically evaluate the literature regarding the use of the neutrophil to lymphocyte ratio (NLR) as a reliable means to detect early PSD development, to help clinicians institute early interventions and improve outcomes.

**Methods:**

Electronic databases, including Web of Science, PubMed, Google Scholar, and Scopus, were searched, and eight studies were included. We assessed the certainty of the associations with GRADE methods.

**Results:**

We found that patients with PSD had higher NLR than the stroke patients with no depression (SMD = 0.51; CI 95% = 0.29-0.73, *p* < 0.001). Also, we found a significantly higher PLR in the patients with PSD when compared to the stroke patients with no depression (SMD = 0.66; CI 95% = 0.19-1.13, *p* < 0.001).

**Conclusion:**

These findings indicated that NLR and PLR could be considered inexpensive biomarkers for the prediction of PSD.

## 1. Introduction

Poststroke depression (PSD) is the most common psychiatric sequela of stroke, affecting about one-third of stroke survivors during the first five years [1, 2]. The presence of depressive episode after stroke diagnosis by physical examination or neuroimaging is associated with a diagnosis of PSD [3]. PSD has been linked to a variety of adverse clinical outcomes, including extended hospital stays, serious functional disability, profound reductions in cognition and quality of life, and inadequate poststroke rehabilitation, and higher mortality [1, 2, 4–6], which all impose a heavy burden on caregivers, healthcare system, and society [7]. This emphasizes the need for early detection of PSD in stroke survivors and identifying people at risk of developing PSD [8].

The complexity of PSD pathophysiology and uncertainty about its predisposing risk factors and underlying biological mechanisms make its prevention, diagnosis, and treatment a complicated task. Thus, the discovery of specific biomarkers at the early stage of stroke is highly demanded because it may assist in predicting PSD [9] and unraveling the pathophysiological mechanisms of PSD, which could lead to the development of specialized targeted treatments [4, 10]. Emerging evidence shows that stroke, depression, and PSD are strongly associated with microglial and astrocyte activation and high levels of proinflammatory cytokines [11]. However, this is critical to detect early as a lot of patients with stroke were previously healthy and nonsymptomatic.

The neutrophil to lymphocyte ratio (NLR) and platelet to lymphocyte ratio (PLR), derived from complete blood count (CBC) differential test, are promising noninvasive prognostic and diagnostic biomarkers in cardiovascular diseases [12–15], cancer [16–18], rheumatologic disease [19], and some neurologic diseases [20–27]. In addition, some previous studies reported significant association of NLR and PLR with PSD, but others did not find any relationship. Although the debate concerning the relationship between these hematological indices and PSD has raged unabated for over a decade, no systematic reviews or meta-analyses have been published. Hence, we performed a comprehensive review and meta-analysis to see if NLR and PLR are linked to PSD susceptibility in patients with stroke, to help clinicians institute early interventions and improve outcomes.

## 2. Methods

### 2.1. Search Strategy

**Figure 1:**
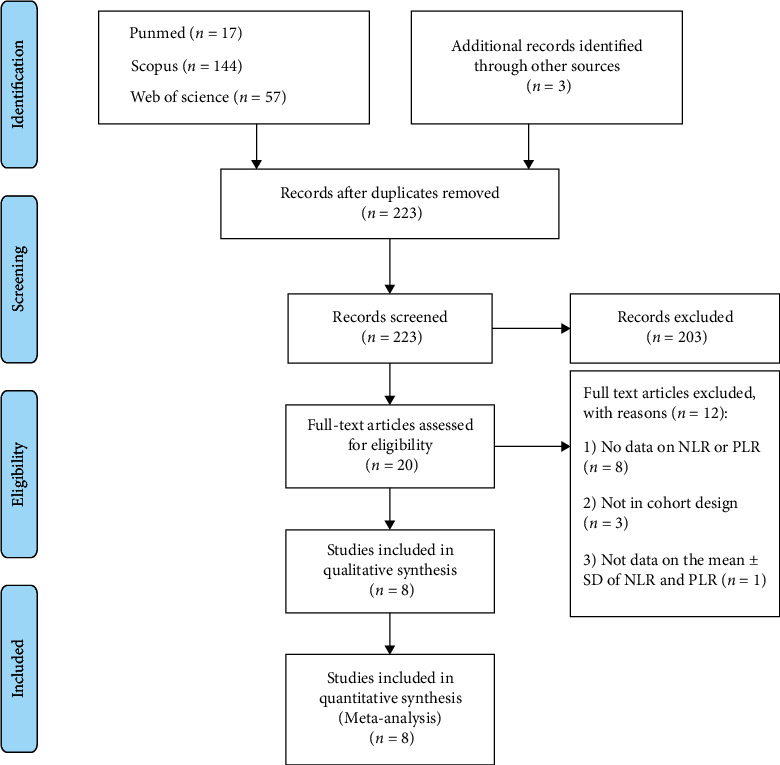
Flow chart of search and study selection.

In compliance with the Preferred Reporting Items for Systematic Review and Meta-Analyses (PRISMA) standards [28], we performed a systematic review and meta-analysis to collect all published papers (Figure 1). No registered review protocol exists.

Two reviewers, who were entirely blind to the author and journal details, separately conducted a systematic literature search in the online databases of PubMed, Web of Science, and Scopus, using the following key words: ((post-stroke depression) OR PSD OR (depression after stroke) OR (depression after cerebrovascular events)) AND ((neutrophil AND lymphocyte AND ratio) OR (neutrophil-to-lymphocyte) OR NLR). The most recent update to the search was on September 14, 2021. We did not limit our search to a specific language or year of release. To find possibly suitable studies, researchers combed through the reference lists of studies found. Additionally, the Prospero Register was combed for information on unpublished and continuing reviews. Because most of the recognized publications were conducted in China, we also conducted a rapid nonsystematic check through Google Scholar as a secondary resource in English and Chinese to uncover grey literature and more relevant studies.

### 2.2. Criteria for Inclusion and Exclusion

Prospective and retrospective cohort studies that evaluated the prognostic role of NLR and PLR in the development of PSD were included. The included articles were checked for adequate and informative data, including the number of subjects in both the PSD and non-PSD (NPSD) groups and mean and standard deviation (SD) of NLR and PLR in both groups. The studies that reported median and interquartile range (IQR) or/and range were also included, and statistical methods were used to estimate mean and SD.

Animal, cross-sectional, and case-control studies, letters to editors, case series, case reports, conference papers, studies with overlapping data, and duplicated studies were excluded.

### 2.3. Extraction of Data

Two authors independently investigated the titles/abstracts of the publications obtained. The full texts of relevant studies independently were checked for eligibility by the same two authors. A third independent author handled any disagreements between authors in both stages.

The following information was extracted: first author, year of publication, study location, study design, age (months), the duration of follow-up, depression scale type, type of stroke (ischemic or hemorrhagic), total sample size as well as number of PSD and NPSD cases, mean and SD of NLR and PLR levels, or any data for estimating the mean and SD (median and IQR or/and range).

### 2.4. Quality Assessment

Two authors independently assessed the quality of the included studies, using the Newcastle-Ottawa scale for cohort studies. The scale has three parts: selection (4 items), comparability (2 items), and outcome (3 items), with a cumulative score of 0 to 9. A leading expert finally settled any differences through arbitration. Studies with scores higher than 7 were considered high quality, those with scores between 5 and 7 were considered moderate quality, and those with scores lower than 7 were considered low quality.

### 2.5. Statistical Analysis

For the NLR and PLR levels, the standardized mean difference (SMD) was provided with a 95 percent confidence interval (CI). The methods introduced by Wan et al. were used to calculate the mean and SD from the median, sample size, range, or IQR [29].

The chi-squared (*χ*^2^) test and the *I*^2^ statistic were used to determine the degree of heterogeneity between study results. Significant heterogeneity of data was defined as *I*^2^ > 75% and *p* < 0.05. In the case of significant heterogeneity, a random effects model was used for meta-analysis; otherwise, we utilized the fixed effects model. Subgroup analysis was performed in the analysis of differences in NLR levels between PSD and NPSD groups according to the duration of follow-up (acute/medium-term phase of stroke (≤1 month) vs. long-term phase (>1 month)), language (Chinese vs. English), and quality of studies (high quality (NOS score > 7) vs. moderate quality (NOS scores 5-7)). Egger's and Begg's tests and the Funnel plot were used to detect potential publication bias, and those with Egger's test *p* < 0.05 were considered significant publication bias. For statistical analysis, Stata 12.0 software (Stata Corporation, College Station, TX, USA) was used. Statistical significance was defined as a *p* < 0.05.

### 2.6. Certainty of Evidence

The certainty of evidence was determined using the GRADE (Grading of Recommendations Assessment, Development and Evaluation) approach by one author for the outcome investigated in meta-analysis (PSD). Finally, the assessments were confirmed by the senior author. According to GRADE, observational studies start at low certainty and may be upgraded for dose-response gradient or for large effect, if suspected biases work against the observed direction of effect, and may be downgraded for publication bias, imprecision, indirectness, inconsistency, and risk of bias.

## 3. Results

### 3.1. Literature Search and Selection

Figure 1 shows the process of identifying and selecting research articles in this systematic review. In addition to 218 studies found from the initial database search, three further studies were identified through reference lists of relevant articles. After removing duplicates, titles, and abstracts, the remaining studies were reviewed, and 20 studies were included for full-text review. Then, 12 studies were excluded (the reasons for exclusion are clarified in Figure 1), and finally, 8 studies were included in the meta-analysis.

### 3.2. Characteristics of the Included Studies

From studies that were included in this meta-analysis, seven studies were in English [20–26] and one in Chinese [27]. Overall, 2194 patients with stroke were enrolled in the selected studies, and 593 patients finally developed PSD. The general characteristics of the included studies are shown in Table 1. Also, the quality assessment of included studies was determined with the Newcastle-Ottawa scale (NOS) (Table 1). Overall, seven studies compared the NLR levels [20–23, 25–27], and three studies determined the PLR levels [20, 23, 24].

### 3.3. Difference in NLR Level in Patients with and without PSD

**Figure 2:**
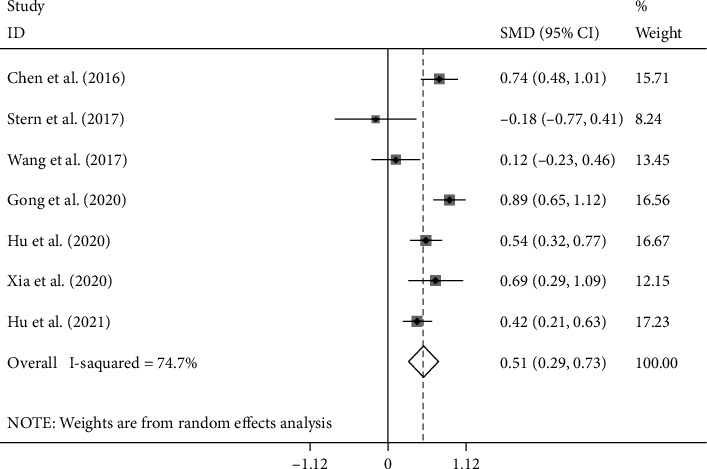
Meta-analysis of NLR levels in PSD and NPSD groups (fixed model).

NLR levels in the PSD group were compared with those of the NPSD group in seven cohort studies [20–23, 25–27] with 1831 patients with stroke, of whom 516 were diagnosed with PSD at the end of the follow-up period. Compared with the NPSD group, NLR levels were significantly higher in the PSD group (SMD = 0.51; CI 95% = 0.29-0.73, *p* < 0.001). The included studies were statistically heterogeneous (*I*^2^ = 74.7%, *p* = 0.001); thus, the random effects model was used for the meta-analysis (Figure 2). However, the certainty of this summary estimate of effect was deemed to be very low using the GRADE approach (Table 2).

**Figure 3:**
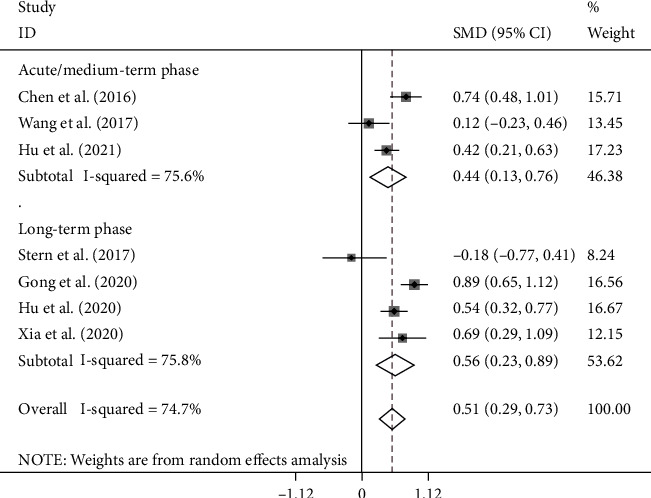
Subgroup meta-analysis of NLR levels in PSD and NPSD groups (random effects model) according to the follow-up period.

In the subgroup analysis according to the follow-up period, three studies assessed the PSD in the acute/medium-term phase of stroke (≤1 month) [21, 23, 26] including 883 patients with stroke of whom 252 developed PSD and four studies concerned the long-term phase and included 948 patients with stroke of whom 264 developed PSD [20, 22, 25, 27]. The NLR levels in patients of the PSD group were significantly more than those of the NPSD group in both the acute/medium-term and long-term phase (SMD = 0.44, CI 95% = 0.13-0.76, *p* < 0.006 and SMD = 0.56, CI 95% = 0.23-0.89, *p* = 0.001, respectively) (Figure 3).

**Figure 4:**
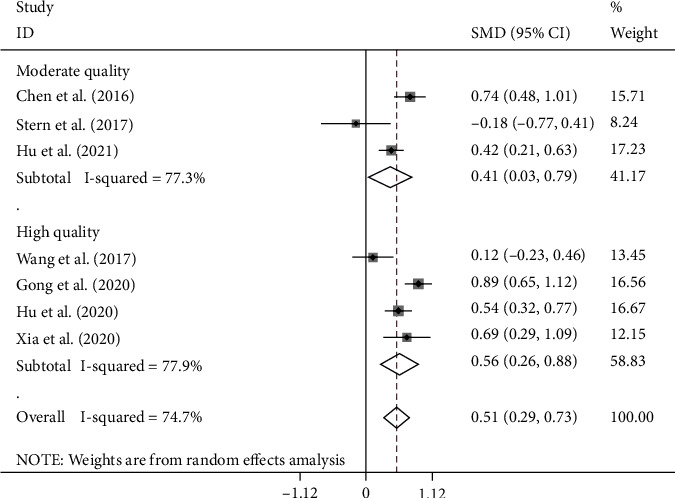
Subgroup meta-analysis of NLR levels in PSD and NPSD groups (random effects model) according to the quality of studies based on the NOS scoring system.

In another subgroup analysis according to the quality of studies, there were four studies with high quality (NOS score > 7), including 1010 patients with stroke of whom 296 developed PSD, and three studies with moderate quality (NOS scores 5-7) included 821 patients with stroke of whom 220 developed PSD. There was no study with low quality. The NLR levels in patients of the PSD group were significantly more than those of the NPSD group in both study groups with high and moderate qualities (SMD = 0.51, CI 95% = 0.29-0.73, *p* < 0.001 and SMD = 0.41, CI 95% = 0.03-0.79, *p* = 0.034, respectively) (Figure 4).

**Figure 5:**
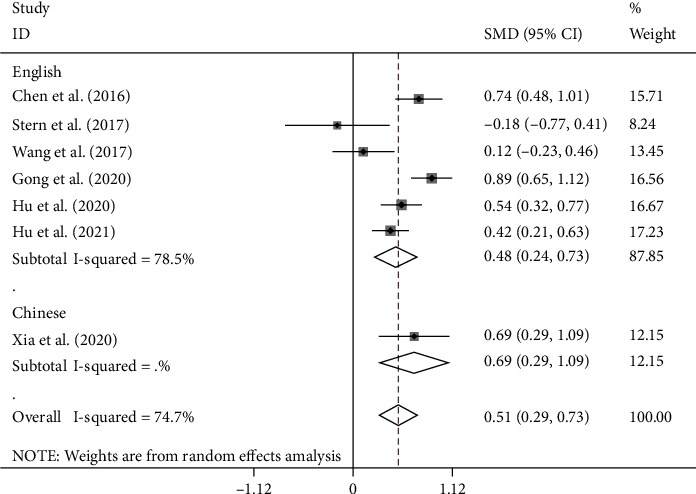
Subgroup meta-analysis of NLR levels in PSD and NPSD groups (random effects model) according to language.

In another subgroup analysis according to language, there were six studies written in English and one study in Chinese. The NLR levels in patients of the PSD group were significantly more than those of the NPSD group in studies written in either English or Chinese (SMD = 0.48, CI 95% = 0.24-0.73, *p* < 0.001 and SMD = 0.69, CI 95% = 0.29-1.09, *p* = 0.001, respectively) (Figure 5).

### 3.4. Differences in PLR Level in Patients with and without PSD

**Figure 6:**
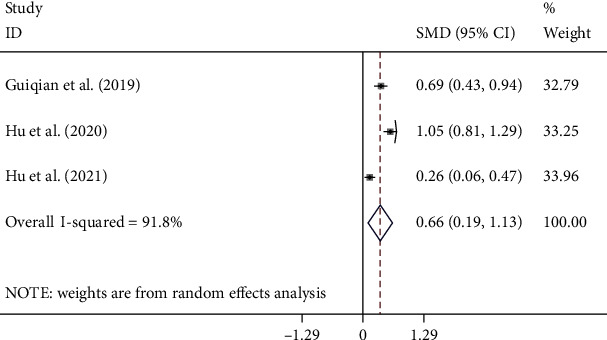
Meta-analysis of PLR levels in PSD and NPSD groups (random effects model).

PLR levels in the PSD group were compared with those of the NPSD group in three cohort studies [20, 23, 24] with 1117 patients with stroke, of which 310 patients were diagnosed with PSD. Compared with the NPSD group, the PSD groups had higher NLR levels (SMD = 0.66, CI 95% = 0.19-1.13, *p* < 0.001). The included studies were statistically heterogeneous (*I*^2^ = 91.8%, *p* < 0.001). Thus, the random effects model was used for the meta-analysis (Figure 6). However, the certainty of this summary estimate of effect was deemed to be very low using the GRADE approach (Table 2). Because of limited studies on PLR, we could not perform any subgroup analysis in this context.

### 3.5. Publication Bias and Small Study Effect

**Figure 7:**
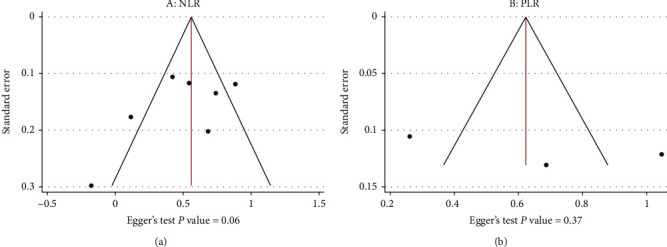
Egger's test and Funnel plot showing publication bias: (a) studies on NLR levels in the PSD and NPSD groups; (b) studies on PLR levels in the PSD and NPSD groups.

Funnel plot and Egger's test were performed to test publication bias. As shown in Figure 7, the results of studies on the prognostic role of neither NLR nor PLR indicated publication bias (Egger's test *p* = 0.06 and 0.37 and Begg's test *p* = 0.08 and 0.54, respectively).

## 4. Discussion

To the best of our knowledge, this is the first time that the prognostic role of NLR and PLR in PSD has been reviewed systematically. Searching main electronic databases for relevant articles, we identified nine studies meeting our inclusion criteria and found two main findings.

We observed that the PSD group had higher NLR levels than the NPSD group (SMD = 0.51; CI 95% = 0.29-0.73, *p* < 0.001). In addition, the PSD group's NLR levels were significantly elevated compared to those of the NPSD group (SMD = 0.66; CI 95% = 0.19-1.13, *p* < 0.001).

Over the past decade, most research in stroke has emphasized the relationship between stroke and inflammation. Stroke, either hemorrhagic or ischemic, leads to a lack of glucose and oxygen, ATP depletion, neuronal death, and inflammation in the nervous system [4].

However, the knowledge about the link between PSD and stroke-induced inflammation is limited [4, 21]. Some researchers suggest that inflammatory mediators change the structure and function of hypothalamic orexin-releasing neurons and decrease the activity and production of monoamine neurotransmitters, such as serotonin, dopamine, and norepinephrine, which may contribute to symptoms of PSD [4, 30, 31]. NLR and PLR as novel inflammatory biomarkers are reported in a variety of neurological disorders. In this study, we found that patients with stroke who developed PSD had considerably higher levels of NLR and PLR than those who did not develop PSD.

The NLR is a practical, low-cost, and easily accessible new inflammatory index [21]. Neutrophils are the earliest part of the immune system to enter the CNS after a stroke [21, 32]. Their activation and recruitment at the site of infarction always coincide with the maximum synthesis of proinflammatory mediators (such as TNF-*α* and IL-6) and associated biomarkers (such as CRP), which result in neuronal damage after stroke [20, 21].

Also, lymphocytes are a type of inflammatory cells with protective and regulatory roles. CD^4+^ T lymphocytes play a key role in preventing poststroke cell injury [21, 23, 32]. Their anti-inflammatory function is linked to the release of anti-inflammatory chemicals (such as TGF-*β* and IL-10). Therefore, high NLR shows high neutrophil and low lymphocyte counts and indicates a high level of systemic inflammation [20, 21, 32]. Since NLR is less likely to be influenced by confounding factors, it may have more predictive value than each parameter on its own [30].

Another low-cost, repeatable, and easily measured index is the PLR. Platelets clump together at the site of injury during a stroke [20, 23, 24]. They are the first-line proinflammatory cells that control parameters like macrophage and neutrophil's recruitment and endothelial permeability [4, 30]. Platelets' dense granules also contain a lot of serotonin and glutamate, which is released as a part of the inflammatory process and could participate in the pathophysiology of PSD [4, 20, 23, 24, 30].

Because the elevated level of NLR and PLR in PSD patients compared to NPSD is solid evidence for the role of inflammation in PSD, anti-inflammatory drugs may be considered potential therapies for PSD [4]. A systematic review published recently found that anti-inflammatory medication, particularly celecoxib, can reduce depression symptoms [33]. In another recent study, a total of 161 individuals with acute ischemic stroke (AIS) were administered with natalizumab, an immunoglobulin against the leukocyte adhesion molecule 4 integrin. The results demonstrated that administration of natalizumab up to 9 h after a stroke contributes to functional and cognitive recovery [34].

### 4.1. Limitations

The findings of this report are subject to some limitations. First, the data extracted from the relevant articles did not permit assessing the relationship between ratios and symptom severity. Second, heterogeneity in studies was greater than expected due to various treatment regimens, duration of recorded stays, center protocols, different study populations, different times of blood tests from which NLR was calculated, and different study designs. Therefore, widespread validity is a concern, and future larger prospective studies are needed. Third, several of the studies are limited by bias whether selection or publication, which should be considered. Fourth, effect size for several of the tests was limited to a few studies. Thereby, widespread adoption and applicability are again a concern warranting further studies. In addition, the study protocol was not prospectively registered. Finally, other biomarker of immune function such as C-reactive protein and cytokines were not assessed; hence, it is impossible to say if elevated NLR and PLR represent an independent marker of immune system abnormalities in PSD patients. Finally, all of the included studies had relatively small sample sizes.

## 5. Conclusion

In conclusion, our meta-analysis provided additional evidence for the role of stroke-induced inflammation in PSD and concluded that NLR and PLR might be used as low-cost predictive biomarkers for PSD. These indicators can b5e extensively employed in the clinic. However, further large-scale and high-quality studies are needed to better understand the link between inflammation and PSD.

## Figures and Tables

**Table 1:** Characteristics of studies included in the meta-analysis.

First author	Study year	Country	Age group (years)	Language	Stroke type	Follow-up period (month)	Depression assessment tool	Sample size		NLR		PLR		NOS score
								PSD	NPSD	PSD	NPSD	PSD	NPSD	
Chen et al.	2016	China	18-80	English	ND	1	HAMD	78	221	3.36 ± 1.13	2.56 ± 1.06	—	—	7
Stern et al.	2017	USA	≥18	English	ICH/IVH	12	HDRS	13	76	6.25 ± 6.44	7.25 ± 5.49	—	—	7
Wang et al.	2017	China	18-80	English	ND	1	HAMD	45	107	2.11 ± 0.93	2.01 ± 0.83	—	—	8
Quiqian et al.	2019	China	ND	English	AIS	1	HAMD	77	286	—	—	131.83 ± 37.17	109.73 ± 30.62	8
Gong et al.	2020	China	≥18	English	ICH	3	HAMD	107	265	6.74 ± 4.24	3.95 ± 2.58	—	—	8
Hu et al.	2020	China	ND	English	AIS	6	HAMD	104	272	3.66 ± 1.61	2.97 ± 1.11	177.03 ± 91.43	115 ± 40.60	8
Xia et al.	2020	China	42-80	Chinese	AIS	3	HAMD	40	70	3.24 ± 1.38	2.38 ± 1.17	—	—	8
Hu et al.	2021	China	18-80	English	AIS	1	HAMD	129	303	2.65 ± 1.53	2.17 ± 0.92	125.01 ± 44.90	114.63 ± 37.19	6

PSD: poststroke depression; NPSD: nonpoststroke depression; NLR: neutrophil to lymphocyte ratio; PLR: platelet to lymphocyte ratio; ND: not declared; HAMD: Hamilton Depression Scale; HDRS: Hamilton Depression Rating Scale; HADS: the hospital anxiety and depression scale; AIS: acute ischemic stroke; ICH: hemorrhagic stroke; IVH: intraventricular hemorrhage; NOS: Newcastle-Ottawa scale.

**Table 2:** GRADE^1^ evidence profile for cohort studies on the role of NLR and PLR in poststroke depression.

Certainty assessment							No. of patients		Certainty^7^	Importance
No. of studies	Study design	Risk of bias^2^	Inconsistency^3^	Indirectness^4^	Imprecision^5^	Publication bias^6^	Participants, *n*	Cases, *n*		
NLR										
7	Observational studies	Not serious	Serious	Not serious	Not serious	None	1831	516	⨁◯◯◯ very low	Critical
PLR										
3	Observational studies	Not serious	Very serious	Not serious	Not serious	None	1117	310	⨁◯◯◯ very low	Critical

^1^Grading of Recommendations Assessment, Development and Evaluation. ^2^Risk of bias based on the Newcastle-Ottawa scale. ^3^When *I*^2^ was <30%, inconsistency was considered not serious limitation; when *I*^2^ > 50, it was considered serious limitation; and when *I*^2^ was more than 75%, it was considered very serious limitation. ^5^Serious limitations were considered when there were fewer than 400 participants for each outcome, and very serious limitations were considered when there were fewer than 300 participants for each outcome. ^6^Funnel plot revealed no asymmetry; neither test of publication bias approached *p* < 0.10. ^7^Data from cohort studies begin with a grade of “low.” Downgraded for very serious inconsistency. ^8^Data from cohort studies begin with a grade of “low.” Downgraded for serious inconsistency.

## Data Availability

All data generated or analyzed during this study are included in this published article.
